# The Effectiveness of the Whole Body Cryotherapy Strategies: A Comparison of Different Duration and Temperature on the Antioxidative Status in the Experimental Rat Model

**DOI:** 10.1155/2019/2065346

**Published:** 2019-05-15

**Authors:** Ewa Romuk, Bronisława Skrzep-Poloczek, Bernadeta Wiśniowska, Aleksander J. Owczarek, Piotr Choręza, Aleksander Sieroń, Ewa Birkner, Dominika Stygar

**Affiliations:** ^1^Department of Biochemistry, School of Medicine with the Division of Dentistry in Zabrze, Medical University of Silesia, Katowice, Poland; ^2^Department of Physiology, School of Medicine with the Division of Dentistry in Zabrze, Medical University of Silesia, Katowice, Poland; ^3^Department of Rehabilitation, 3rd Specialist Hospital in Rybnik, Rybnik, Poland; ^4^Department of Statistics, Department of Instrumental Analysis, School of Pharmacy with the Division of Laboratory Medicine in Sosnowiec, Medical University of Silesia, Katowice, Poland; ^5^Department of Clinical Internal Medicine, Angiology and Physical Medicine, School of Medicine in Zabrze, Medical University of Silesia, Katowice, Poland

## Abstract

**Background:**

We examined the effectiveness of the systemic cryotherapy in terms of the temperature and duration of the therapeutic series measured by oxidative stress markers in the rat animal model.

**Methods:**

Antioxidants in serum, plasma, liver, and erythrocytes were evaluated in two study groups following 1 min exposure to − 60°C and − 90°C, for 5 and 10 days.

**Results:**

Superoxide dismutase activity in the tissues was lower than in the serum. The glutathione peroxidase was significantly higher in − 60°C than in − 90°C, in both 5 and 10 days of exposition. The liver catalase CAT were significantly lower in − 60°C when compared to − 90°C for 5 and 10 sessions of exposure. In all analysed tissues, the sessions of cryotherapy, – 60/5 and – 60/10, were more effective in reduction malondialdehyde than sessions of − 90/5 and − 90/10. The highest total antioxidant capacity was observed in the − 60/5 group.

**Conclusions:**

Whole body cryotherapy based on temperature − 60°C may be considered as more beneficial than − 90°C for most of the oxidative stress (OS) markers measured in the selected tissues. The temp. − 60°C is more beneficial than − 90°C when measured by activity of Total SOD, CAT, and GPx. The therapeutic sessions − 60/10 and − 60/5 were the optimal schemes of WBC model in terms of TAC and MDA amount.

## 1. Introduction

Whole body cryostimulation (WBC) is becoming a popular method used in sport and medicine in order to achieve the therapeutic results by stimulation of numerous clinical, physiological, and biochemical processes [1–3]. Dependent on the clinical and therapeutic aims, very low temperatures ranging from – 100°C to – 160°C over a short time span from 1 up to 4 min are used in order to provoke the systemic response including antioxidant systems, cardiovascular system, anti-inflammatory, and analgesic processes as well as lower of muscle tension, increase the stabilization of lysosomal membranes, and increase secretion of selected hormones [4–9]. The changes in the cell metabolism during WBC may lead to an increase in the oxidative stress (OS) by the intensification of the reactive oxygen species (ROS) production and reactions [10]. Oxidative stress is a hallmark of many chronic diseases and after the injury but the direct estimation of reactive oxygen species (ROS) generation in vivo is difficult; thus currently and widely the enzymatic antioxidative and antioxidative systems are used in order to assess the effects of WBC [11, 12]. The mechanism which leads to pain release, inflammatory symptom alleviation, and recovery improvement after physical injury and in chronic diseases seems to be related to cold-induced analgesia and cold-induced lower levels of oxidative stress and inflammation [13].

Despite a growing interest in cryostimulation, there are only a few experimental studies which provide clear data on the effects of a different range of extremely low temperatures and different time expositions on a subjected healthy individuals and selected metabolic pathways [14]. There are also very limited studies comparing the effects of a different number of WBC, or regarding exposure protocols and the relationship between studied parameters such as temperature, duration of the exposition in minutes, a number of repetitions, and the treatments' expected effects [13]. In practice, the protocols of the cryotherapies differ in a type of cryochamberer used, the temperature applied, the duration of the sessions, and the number of therapeutic cycles used [12, 14–16]. The selection of the cryotherapy parameters is frequently based on the recommendations of cryochamber manufacturers, many times without a rational and scientific base. In order to increase the practical knowledge of the beneficial and harmful spectra of costimulation, the detailed research in that matter is necessary. Thus, the aim of the study was to validate the effectiveness of the systemic cryotherapy in terms of the temperature and duration of the therapeutic series measured by enzymatic and nonenzymatic oxidative stress markers in the rat animal model.

## 2. Materials and Methods

### 2.1. Animals

Animal care was performed according to previously described procedures [17]. Wistar FL rats (12-week-old males, weight 300–470 g) came from the Center of Experimental Medicine of the University of Silesia in Katowice, Poland. All animals were housed and maintained in the same conditions (22 ± 1°C, humidity 60%  ± 5, and 12-hour light-dark cycles), with a standard diet and water ad libitum. All experimental procedures were conducted according to the ethical standards and protocols approved by the Local Ethics Committee of the Medical University of Silesia in Katowice, number 29/01. The study was performed in accordance with principles and guideline for the care and handling of laboratory animals.

### 2.2. Experimental Model

**Figure 1:**
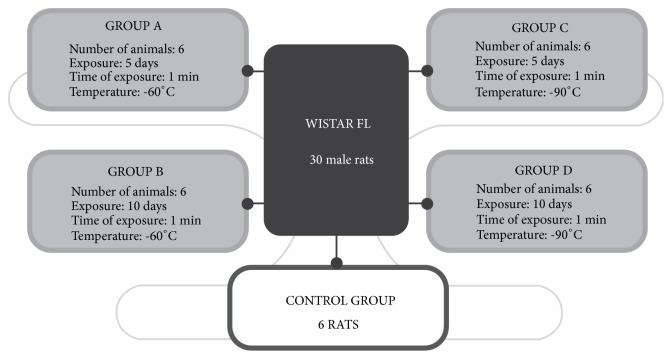
Scheme of the study groups.

The design of the experiment was conducted according to the previously reported methodology [17]. Rats were randomly divided into 4 groups and control (*n *= 6, Figure 1). All experimental groups and the control group were treated in the same way with one exception that control rats were not exposed to low temperatures. The endpoints to all animals were the same in terms of time and sampling. The animals were exposed to − 60°C or − 90°C for 1 minute in a cryogenic chamber for 5 and 10 consecutive days; these exposure conditions are denoted as − 60/5, − 60/10, − 90/5, and − 90/10, respectively. The temperature and time of exposure were assessed during a pilot study. The conditions applied were safe for animals and caused no harm or side effects (Figure 1).

### 2.3. Cryotherapy

The cryochamber (type Wroclaw, Zagrobelny-Raczkowski-Strank, Wrocław, Poland) was divided into two compartments held at different temperatures (− 60°C and − 90°C) measured before cryostimulation and at the level of animals. The animals were weighed daily before WBC. The air in both chambers contained 22% oxygen and 78% nitrogen. The walls of the chamber were lined with multilayer thermal insulation for use at low temperatures. The cryochamber was equipped with three entrances. The doors had a window, which allowed for the visual inspection of the procedure. The humidity and temperature were monitored by an operating system. The rats were exposed to temperatures − 60 and − 90°C using the two-stage cryogenic chamber every day at the same time between 9 am and 10 am for 5 and 10 consecutive days (Figure 1). During exposition to very low temperature in cryochamber rats were kept in wooden cages placed on the floor. There were the following study groups: − 60/10, − 60/5, − 90/10, and − 90/5, corresponding to the temperature (− 60°C and − 90°C) and number of 1 min sessions applied (5 or 10). The eating behavior and physical activity were controlled daily. There were no signs of complications after cryotherapy sessions in all subjected animals.

### 2.4. Sample Collection and Tissue Preparation

After 5- and 10-day WBC periods, anesthesia was induced with the use of isoflurane 2% and oxygen flow at 2 L/min under spontaneous breathing. Blood (3–5 mL) samples were collected from the right ventricular via tubes with and without EDTA. Samples were collected after centrifugation at 4000 rpm for 10 minutes at 4°C; the serum and plasma was subsequently snap frozen in liquid nitrogen and stored at − 80°C until analysis. The erythrocytes were washed three times with buffered NaCl solution (PBS: 0.01 mol phosphate buffer 0.14 mol NaCl, pH 7.4), chilled to 4°C, and finally snap frozen in liquid nitrogen and stored at − 80°C until analysis. The erythrocytes were thawed before analysis, and the haemolysate of the washed red blood cells was diluted with distilled water and chilled to 4°C. The liver was perfused with 0.9% NaCl and tissues were harvested and washed using 0.9%. NaCl. The tissue was homogenized (1: 10w/v) in 0.9% NaCl (homogenizer Potter-Elvehjem PTFE pestle and glass tube, Sigma-Aldrich) and then centrifuged (10 min, 4000 rpm, 4°C). Homogenates were snap frozen in liquid nitrogen and stored at – 80°C until further analysis, for no longer than 30 days.

### 2.5. Protein Concentration

Protein concentration was determined by Lowry methods [18].

### 2.6. Oxidative Stress Marker Analyses

Analysis of antioxidant system parameters were done by determining the activity of antioxidant enzymes: total superoxide dismutase activity (SOD), catalase (CAT), glutathione peroxidase (GPx), glutathione transferase (GST), and glutathione reductase (glutathione-disulfide reductase, GR, GSR); nonenzymatic antioxidant system: the total antioxidant capacity (TAC), and the lipid peroxidation by determining the concentration of malonic dialdehyde.

### 2.7. Superoxide Dismutase Analysis (EC 1.15.1.1)

The Total SOD activity was determined in blood serum, haemolisates, and liver tissue with the use of the spectrophotometric method by Oyanagui [19]. Enzyme activity was expressed as nitrite units (NU) per mL serum, (NU) per mg haemoglobin, and (NU) per mg protein. One NU exhibits 50% inhibition of the formation of a nitrite ion under the method's condition.

### 2.8. Catalase Activity (EC 1.11.1.6)

The catalase activity was measured in erythrocytes and liver homogenates using kinetic method by Aebi. The enzymatic activity was expressed in IU/g Hbg and IU/mg protein [20].

### 2.9. Glutathione Peroxidase Activity (EC 1.11.1.9) 

GPx activity in the haemolisates and liver homogenates was measured by incubation with GPx buffer (100mM potassium phosphate with 1 mM EDTA pH7.7), 40 mM sodium azide, GSH (diluted in 5% metaphosphoric acid), GR (GPx diluted in buffer), NADPH (diluted with sodium bicarbonate 5%), and 0.5mM tert-butyl. The decay of NADPH concentration was evaluated for 10 minutes in a spectrophotometer, at 340 nm [21].

### 2.10. Transferase Glutathione-S Activity (EC 2.5.1.18)

GST activity was determined in the haemolisates and liver homogenates by the kinetic method of Habig and Jakoby [22]. 1-Chloro-2,3-dinitrobenzene was used as a substrate and results are expressed in IU/g protein and IU/mg Hbg.

### 2.11. Glutathione Reductase Activity (EC 1.8.1.7)

The enzymatic activity of GR in the haemolisates and liver homogenates was measured by a decrease in the concentration of NADPH in the samples using GR buffer (200mM sodium phosphate pH7.5, 6.3mm EDTA). The kinetic reading was performed at a wavelength of 340 nm for 10 minutes [23].

### 2.12. Total Antioxidant Capacity (TAC)

TAC was measured using a commercial kit (Randox Co., England). The 2,2′azino-di-(3-ethylbenzothiazoline sulphonate) (ABTS) was incubated with a peroxidase (metmyoglobin) and hydrogen peroxide to produce the radical cation ABTS+ which has a relatively stable blue-green color and measured at 600 nm. The suppression of the color was compared to the standard for TAC measurement assays (Trolox). The assay results are expressed as Trolox equivalent (mmol/L).

### 2.13. Lipid Peroxidation

Plasma, haemolysates, and liver MDA concentration was measured according to the Ohkawa et al. method using the reaction with thiobarbituric acid with spectrophotometric detection employing 515 nm excitation and 552 nm emission wavelengths. MDA concentration was calculated from the standard curve, prepared from 1,1,3,3-tetraethoxypropane [24].

## 3. Statistical Analysis

Statistical analysis was performed using STATISTICA 10.0 PL (StatSoft, Cracow, Poland) StataSE 12.0 (StataCorp LP, TX, US). Statistical significance was set at a* p*-value below 0.05. All tests were two-tailed. Interval data were expressed as mean value ± standard deviation in the case of a normal distribution or as median/interquartile range in the case of data with skewed or nonnormal distribution. Distribution of variables was evaluated by the Shapiro-Wilk test and homogeneity of variances was assessed by the Levene test. For comparison of data, the two-way ANOVA analysis was used with post hoc contrast analysis. In the case of skewed data distribution logarithmic transformation was done before analysis.

## 4. Data Analysis

**Figure 2:**
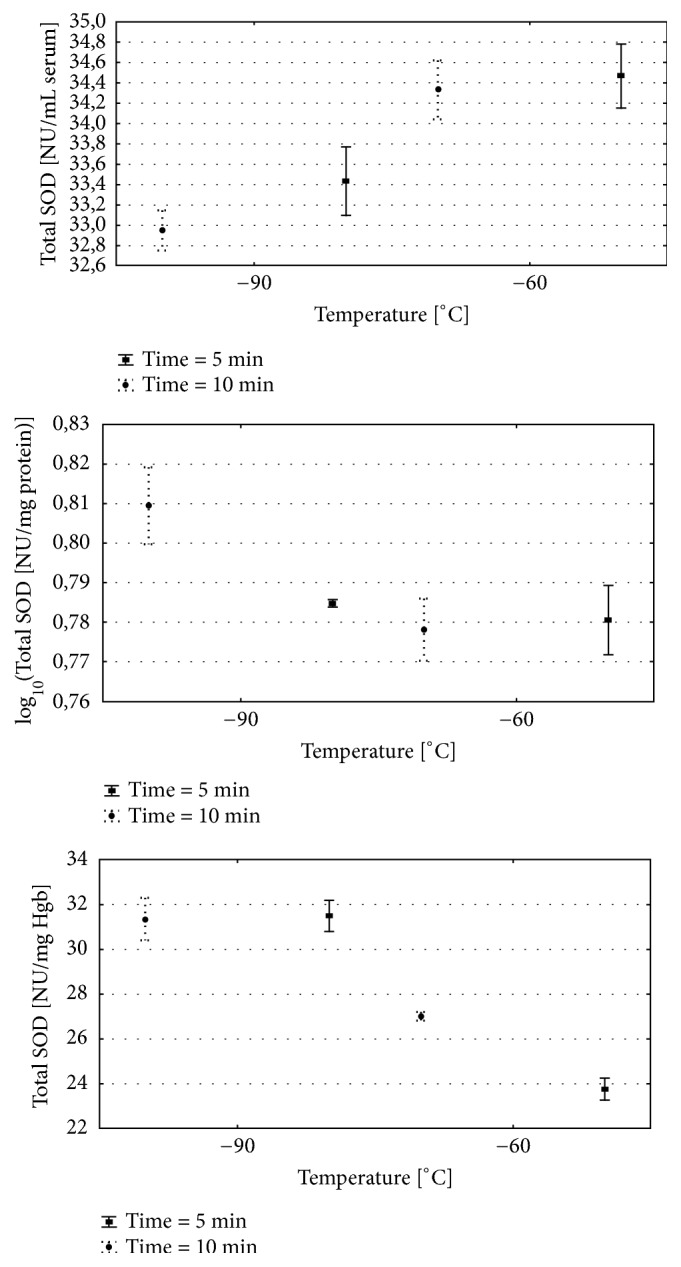
(a) Serum Total SOD activity for various WBC procedures. (b) Erythrocytes Total SOD activity after for various WBC procedures. (c) Liver Total SOD activity for various WBC procedures. Group − 60/5:1 min of WBC stimulation, 5 days, − 60°C; group − 60/10: 1 min of WBC stimulation, 10 days, − 60°C; group − 90/5: 1 min of WBC stimulation, 5 days, − 90°C; group − 90/10: 1 min of WBC stimulation, 10 days, − 90°C. Vertical lines depict 95% confidence interval. Statistical significance was set at a* p*-value below 0.05.

**Figure 3:**
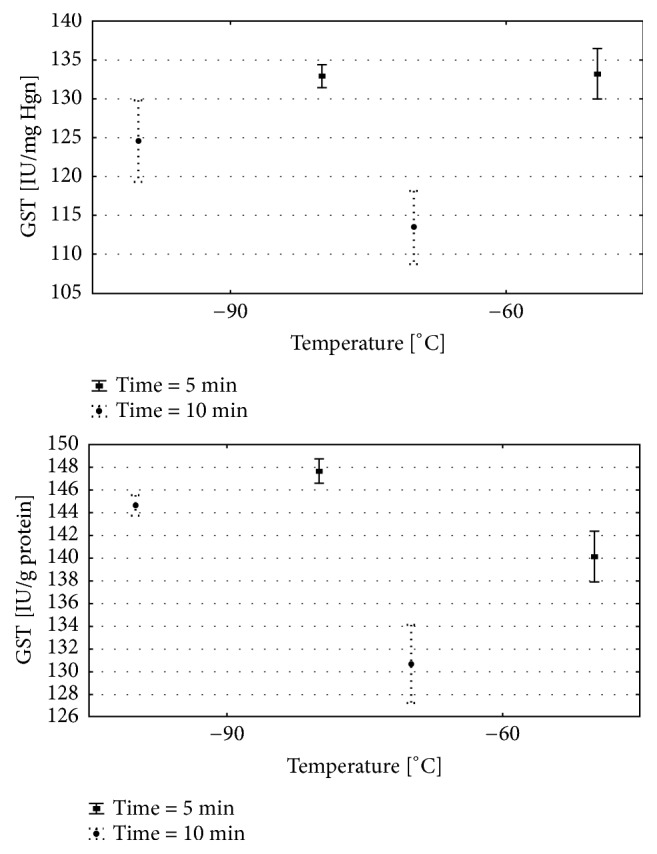
(a) Erythrocytes GST activity for various WBC procedures. (b) Liver GST activity for various WBC procedures. Group − 60/5:1 min of WBC stimulation, 5 days, − 60°C; group − 60/10: 1 min of WBC stimulation, 10 days, − 60°C; group −90/5: 1 min of WBC stimulation, 5 days, − 90°C; group − 90/10: 1 min of WBC stimulation, 10 days, − 90°C. Vertical lines depict 95% confidence interval. Statistical significance was set at a* p*-value below 0.05.

**Figure 4:**
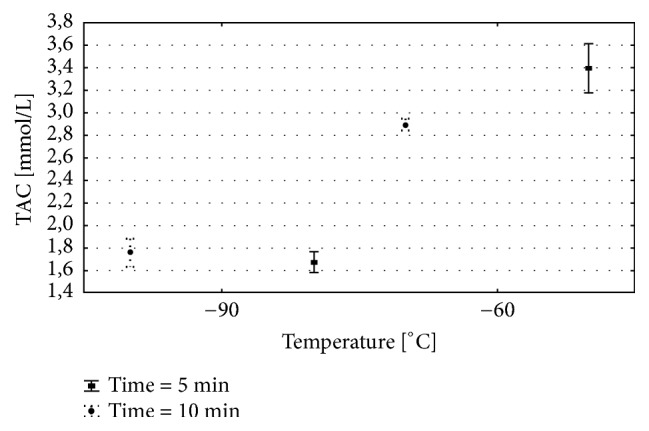
The concentration of total antioxidant capacity (TAC) in plasma for various WBC procedures. Group − 60/5:1 min of WBC stimulation, 5 days, − 60°C; group − 60/10: 1 min of WBC stimulation, 10 days, − 60°C; group − 90/5: 1 min of WBC stimulation, 5 days, − 90°C; group − 90/10: 1 min of WBC stimulation, 10 days, − 90°C. Vertical lines depict 95% confidence interval. Statistical significance was set at a* p*-value below 0.05.

The two-way analysis of variance was done for each of the analysed variables. Detailed results are presented in Table 1 and on Figures 2–4. Results of multiple comparisons are presented in Table 2.

## 5. Results

### 5.1. Total SOD Activity

Total SOD serum: the time of exposure and temperature significantly influenced the activity of Total SOD (Table 1). Total SOD activity was significantly higher after 5 and 10 sessions of 1 min exposure to − 60°C when compared to − 90°C groups (Figure 2(a); Table 1). The SOD activity was significantly different when compared to − 60/5 vs. − 90/5 and − 60/10 vs. − 90/10 (Figure 2(a), Table 2). Nevertheless, for the temp. − 60°C there was no significant difference in the Total SOD activity between the numbers of sessions 5 vs. 10 (Table 2). There were significant differences in serum Total SOD activity between − 90/5 and − 90/10 experimental groups (Table 2). After 10 therapeutic sessions in the − 90°C the Total SOD activity was significantly lower when compared to 90/5 group (Figure 2(a); Tables 1 and 2).

Total SOD Hgb: the Total SOD activity measured in haemolisate was influenced by time, temperature, and interaction between temperature and time. The activity of Total SOD was significantly higher in haemolisate of animals exposed to − 90/5 and − 90/10 when compared to − 60/5 and − 60/10 (Figure 2(b); Tables 1 and 2). In group exposed to − 60°C a significant increase in Total SOD activity was observed after 10 cryosessions in comparison to 5 cryosessions. There were no differences between Total SOD activity measured in − 90/10 and − 90/5 observed (Figure 2(b); Tables 1 and 2).

Total SOD liver: the Total SOD activity measured in liver tissue was influenced by time, temperature and interaction between temperature and time. The highest mobilisation of Total SOD activity was observed in group − 90/10 when compared to other study groups (Figure 2(c); Table 1). The temperature had the significant impact on the Total SOD in case of 10 days of sessions. The significant differences between − 60/10 and 90/10 groups were observed. The Total SOD activity was significantly higher in − 90/10 in comparison to − 90/5 (Figure 2(c); Tables 1 and 2).

### 5.2. GPx Activity

GPx Hgb: GPx activity was significantly influenced by numbers of sessions, temperature, and interactions between those two parameters. GPx activity was significantly higher in the groups exposed to 5 and 10 WBC sessions in − 60°C when compared to − 90°C (Tables 1 and 2).

GPx liver: GPx liver activity was significantly influenced by numbers of sessions, the range of temperature, and interactions between those two parameters. The GPx activity was significantly higher in − 60/10 and − 60/5 when compared to − 90/5 and − 90/10 groups (Tables 1 and 2). There were no significant differences in the therapeutic effects between − 60°C, 5 and 10 sessions (Table 2). The significant differences in the GPx activity were observed between the number of sessions in − 90°C. After − 90/10 WBC sessions, the GPx activity significantly increased in comparison to − 90/5 (Table 2).

### 5.3. CAT Activity

CAT Hgb: the Hgb CAT activity was influenced by time of exposure (numbers of sessions), the range of temperature, and interaction between analysed parameters. Hgb CAT activity was significantly higher after 5 and 10 WBC sessions when compared to − 60/10 and − 60/5 (Tables 1 and 2).

After 10 days of 1 min exposure to − 90°C a significant decrease in CAT activity compared to 5 days was observed (Tables 1 and 2). That may suggest the depletion of the Hgb CAT activity and rather an unfavourable effect to intensive WBC in terms of time of exposure measure be the number of sessions and the range of temperature.

CAT liver: the CAT activity was assessed in the liver tissue related to the time of exposure, temperature, and interaction between those parameters (Table 1). The exposition to the temp. − 90°C caused a significant increase in liver CAT activity when compared with temp. − 60°C exposition in both groups after 5 and 10 days exposition (Tables 1 and 2). The activity of CAT in the liver after 10 days of exposition in − 90°C was significantly higher than activity after 5 days of exposition (Tables 1 and 2).

### 5.4. GST Activity

GST Hgb: the GST Hgb activity was related to time, temp., and its interaction (Table 1). After 5 sessions of cryotherapy GST activity in red blood cells was similar in the groups exposed to − 60°C and − 90°C (Figure 3(a); Tables 1 and 2). However, after 10 days of exposure, Hgb GST activity was significantly increased in the group exposed to − 90°C in comparison to − 60°C. At both temperatures used, after 10 days of exposure, a significant decrease in GST activity occurred compared to the group exposed for 5 days (Figure 3(a); Tables 1 and 2).

GST liver: the liver GST activity was also dependent on the time of exposure, temperature, and interactions of these parameters (Table 1).

The liver GST activity significantly differs in all analysed groups. The highest values of GST were observed in groups exposed to 5 WBC sessions in − 60°C and − 90°C when compared to 10 WBC sessions (Figure 3(b); Tables 1 and 2). Nevertheless, the GST activity was significantly increased in the group exposed to − 90°C when compared to − 60°C, after both 5 and 10 WBC sessions.

### 5.5. GR Activity

GR Hgb and liver: the GR Hgb activity was influenced by the interaction between time of exposure (the number of 1 min sessions, Table 1).

GR liver: the GR in liver tissue activity was influenced by the numbers of sessions and the range of temperature (Table 1). The GR activity was comparable after 5 sessions of WBC in − 60°C and − 90°C (Tables 1 and 2). GR activity significantly increased after 10 sessions in WBC in − 90°C when compared to WBC therapeutic sessions conducted in − 60°C (Tables 1 and 2).

### 5.6. MDA

MDA plasma: for all analysed tissues the significant differences in MDA concentration were observed only in the relation to time (Table 1). Significantly lowered MDA concentrations were observed in the groups treated with − 60°C when compared to − 90°C for 10 min, which we consider to be a beneficial effect of the lower temperature.

MDA Hgb: lipid peroxidation measured by MDA concentration in the haemolysate was significantly different in relation to time and temperature (Table 1). Significantly lowered MDA concentration was detected in the tissue collected from animals subjected to the − 60/5, − 60/10 procedure in comparison to − 90/5 and − 90/10. The numbers of days of cryotherapy with − 60°C were more beneficial than − 90°C (Tables 1 and 2).

MDA liver: time and temperature significantly influenced the MDA concentration levels in the liver tissue (Table 1). The MDA concentration was significantly lower in the group − 60/5 when compared to − 90/5 (Tables 1 and 2). Longer duration of WBC procedure in the − 90/10 group significantly decreased MDA concentration in relation to − 90/5 (Tables 1 and 2).

### 5.7. Total Antioxidant Capacity

TCA serum levels were influenced by the numbers of sessions, temperature, and interactions between those parameters. The significantly higher TAC amount was observed in − 60/10 and − 60/5 therapeutic model when compared to − 90/10 and − 90/5. For that parameter, the optimal model was − 60/5 where the TAC amount was the highest among all analysed groups. Very low temperature − 90°C leads to significant decrease in the serum TAC amount for 5 and 10 WBC sessions when compared to − 60°C (Figure 4; Tables 1 and 2).

## 6. Discussion

The whole body cryotherapy is more often used in modern rehabilitation for treatment of various diseases, to enhance recovery after exercise and facilitate recovering after injury [13, 15, 25, 26]. Nevertheless, there are very few studies available, which concern the optimal conditions of WBC in terms of a range of temperature, duration of the exposition, and its physiological outcomes. Thus, in the present study, we tested two WBC protocols, which differ in terms of a number of cryosessions and range of therapeutic temperature in the rat animal model. After 5 and 10 sessions of 1-minute cryostimulation in − 60°C and − 90°C we can conclude that (i) the impact of number of sessions was very subtle, and varied in different tissues; (ii) analysed enzymes are sensitive markers of exposure to the extreme cold and its activity depends on the temperature and number of sessions. Most of the selected parameters depended equally on the temperature, number of sessions, and interactions between those parameters; (ii) despite the number of sessions applied, the Total SOD activity measured in the tissues was lower than measured in the serum; (iii) the GPx activity was significantly higher after exposition to higher − 60°C rather that to − 90°C, in both tested numbers of sessions (5 and 10); (iv) the CAT activity measured in the tissues of red blood cells and liver was significantly lower in the temperature of − 60°C when compared to − 90°C for both analysed types of therapeutic schemes (5 and 10 sessions of exposure); (v) the GST is a sensitive parameter, and its activity depends both of temperature and number of sessions; (vi) in all analysed tissues, the sessions of cryotherapy with − 60°C during 5 and 10 days may be considered to be more effective in reduction of oxidative stress measured by MDA concentration than sessions and series of − 90/5 and − 90/10; (vi) the highest TAC was observed in the group of animals subjected to the 5 sessions of cryotherapy in the temperature of − 60°C.

The adaptation of animals to the low temperature is associated with increased production of ROS by mitochondria and peroxisomes [27]. Well-known and commonly used markers of oxidative stress are Total SOD, GPx, and CAT that are the first line of defense against free radicals [28]. In this study, the time of exposure and the range of temperature had a significant impact on the activity of selected antioxidative enzymes, MDA and TAC concentrations. The mobilisation of Total SOD activity was lower in − 60°C for two of three selected tissues, what may be considered as less harmful effect in terms of induction of OS. The lower temperature − 90°C and the higher number of 10 sessions increased Total SOD activity in red blood cells and liver. The GPx activity significantly decreased after 10 WBC sessions when compared to 5 WBC sessions for both − 60°C and − 90°C. The changes in enzymes activity in selected study groups may be an effect of the adaptation to the cryostimulation or depletion of enzymes efficiency due to too long exposition to the general stressor which can be a WBC. Lubkowska and coauthors presented the study where the single cryostimulation induced an increase in GPx plasma concentration, but the treatment of 10 repeated cold sessions, lasted 3 min, lead to the decrease in the total oxidative and antioxidative status [29]. A different form of exposure of the whole body to the cold, a winter swimming in ice-cold water, also leads to oxidative stress and a higher baseline levels of SOD, CAT and GPx reducing enzymes in erythrocytes when compared with control subjects [30, 31]. In this study, the higher number of the WBC sessions (10) in − 60°C and − 90°C leads to the significant decrease in GST Hgb and liver activity when compared to 5 WBC sessions. The increase in CAT and GST activity in – 90/5 and – 90/10 exposition group in comparison with – 60/5 and – 60/10 groups may be the result of the activation of defense mechanisms aimed at balancing the increased production of free radicals as the increased lipid peroxidation observed as higher MDA and lower TAC in – 90/5 and – 90/10 groups. The decrease in – 90/5 and – 90/10 liver Total SOD activity and the decrease in liver and haemolisate – 90/5 and – 90/10 GPx activity may be due to the depletion of these enzymes and its reduced synthesis. Additionally, GPx may be inhibited by an excess of superoxide radical which is a substrate for Total SOD. Miller et al. hypothesized that the generation of ROS may be partly inhibited by ten sessions of WBC due to the increase of superoxide dismutase (SOD), uric acid (UA), and total antioxidant status in plasma (TAS) in multiple sclerosis patients and healthy patient [12, 32]. Lubkowska and coauthors conducted a very interesting study, where assessed the effect of 3 minutes to cryogenic temperatures (− 130°C) for 20 consecutive days in healthy subjects. After 20 sessions of WBC, the activity of SOD in erythrocytes significantly increased in comparison to 10 WBC sessions but the activity of CAT, GPx, and glutathione concentration, as well as oxidized glutathione (GSSG) concentration, significantly decreased in comparison to 10 WBC sessions. The authors suggested that some of these changes may depend on the body mass index of participants [29]. Other studies, presented by Woźniak et al., confirmed that the WBC per se stimulates the generation of ROS and leads to a disturbance in the prooxidant-antioxidant balance. Nevertheless, oxidative stress induced by WBC triggered adaptation changes that protected highly trained kayakers against a disturbance in the prooxidant-antioxidant balance during physical training [33]. Taking into consideration enzymatic activity of assessed parameters in this study, we suggest more beneficial effect of WBC sessions set in – 60/5 and – 60/10 when compared to − 90°C.

When analysing MDA concentration in serum, erythrocytes, and liver of the − 60°C exposition group we have observed significant increase only in liver tissue after 10 days exposition versus 5 days. In − 90°C group the significant increase in MDA concentration in erythrocytes and decrease in liver MDA concentration after 10 days exposition versus 5 days were observed. In this study, the response of liver tissue to selected cryotherapy procedures was more subtle, and every studied parameter caused significant change in liver MDA concentration. Nonetheless, we can conclude that the most beneficial for reduction of lipid peroxidation was series of higher temperature − 60/5, − 60/10 and series of very low temperatures and high number of sessions − 90/10, what probably may be understood as an effect of an adaptive and protective mechanism. TAC is described as the cumulative action of all the antioxidant agents present in the selected tissue; thus it is understood as an integrated parameter rather than the simple sum of measurable antioxidants, strongly modified by OS [34–36].

In the presented study − 60/10 and − 60/5 were also the optimal schemes of WBC model in terms of TAC. Sutkowy and colleagues studied highly trained kayakers subjected to 2 sessions of WBC (− 140°C) per day, during 10 days of intensive training. The presented results show that extremely low temperatures and physical exercise merged together helped to maintain the oxidant/antioxidant balance increasing [37]. Miller et al. showed no increase in TBARS in plasma after 10 sessions of WBC treatment in healthy men [12]. Stanek et al. observed a significant decrease in MDA concentration in serum and erythrocytes of male patients with active-phase ankylosing spondylitis and decrease in other markers like oxLDL and antibody anti-oxLDL, TOS, and OSI during WBC in − 120/10 days [38]. The effect of WBC on the oxidant/antioxidant balance depends on the therapeutic design of the studies and characteristics of the study groups: healthy individuals, sportsman, or patients. Most of the WBC studies report the beneficial effect of the extreme cold on the antioxidant/prooxidant balance and antioxidant capacity of the organism. Nonetheless, WBC itself triggers increased production of ROS and oxidant/antioxidant imbalance which is not emphasized. This phenomenon can be explained by the hormesis theory when a stressor can have a tempering effect if it is used regularly for a longer period at optimal intensity [25, 37]. In the therapeutic practice, the most common application of cryostimulation is a series of 10 WBC sessions, without providing any scientific justification [29]. It is also proved that the favorable adaptive changes in the activity of antioxidant enzymes and lipid profile may appear after a long series of WBC, even 20 sessions [29]. Taking into consideration the results of this study we suggest that WBC used in moderated, an optimal range of time and exposure to the temperature stimulate the antioxidant systems; nevertheless if the stimulation is too intensive or lasts too long, it leads to the depletion of the antioxidant defense reducing the positive impact of WBC.

## 7. Conclusions

Taking into consideration all analysed parameters of antioxidative systems in serum, erythrocytes, and liver tissue, the therapeutic scheme of WBC based on higher temperature − 60°C may be considered as more beneficial than lower − 90°C for most of the oxidative stress (OS) markers measured in the selected tissues. The temperature − 60°C is more beneficial than − 90°C when measured by activity of Total SOD, CAT, and GPx. The therapeutic sessions − 60/10 and − 60/5 were the optimal schemes of WBC model in terms of TAC and MDA amount.

The main limitation of this study is a small number of animals used in the experiment and some heterogeneity observed in the individual, biological answers observed in the studied subjects.

## Figures and Tables

**Table 1:** Enzymatic and nonenzymatic antioxidant systems in serum, haemolisates, and liver tissue of rats after WBC. The table presents descriptive statistics and results of two-way analysis of variance.

Temp.:	60		90		ANOVA		
Time:	5	10	5	10	p_Time_	p_Temp._	p_Inter._

Total SOD [NU/mL]	34.47 ± 0.30	34.33 ± 0.27	33.43 ± 0.32	32.95 ± 0.19	**< 0.001**	**< 0.05**	0.135

Total SOD [NU/mg Hgb]	23.73 ± 0.47	27.00 ± 0.15	31.50 ± 0.67	31.33 ± 0.91	**< 0.001**	**< 0.001**	**< 0.001**

Total SOD [NU/mg protein]	6.08 (6.05 – 6.09)	6.02 (6.01 – 6.05)	6.09 (6.08 – 6.10)	6.45 (6.30 – 6.60)	**< 0.001**	**< 0.01**	**< 0.001**

GPX [IU/g Hbg]	154.0 ( 154.0 – 157.0)	140.0 (139.0 – 141.0)	88.4 (88.3 – 88.6)	63.7 (63.5 – 93.9)	**< 0.001**	**< 0.001**	**< 0.001**

GPX [IU/mg protein]	9.93 ± 0.16	9.83 ± 0.08	5.95 ± 0.64	6.93 ± 0.32	**< 0.001**	**< 0.05**	**< 0.01**

CAT [IU/g Hbg]	403.0 ± 13.5	408.8 ± 9.2	539.2 ± 15.1	513.0 ± 8.6	**< 0.001**	**< 0.05**	**< 0.01**

CAT [IU/mg protein]	99.1 ± 0.5	96.2 ± 1.45	221.5 ± 6.2	235.0 ± 4.3	**< 0.001**	**< 0.01**	**< 0.001**

GST [IU/mg Hbg]	133.3 ± 3.3	113.5 ± 4.5	133.0 ± 1.4	124.7 ± 5.0	**< 0.01**	**< 0.001**	**< 0.01**

GST [IU/g protein]	140.2 ± 2.1	130.7 ± 3.3	147.7 ± 1.0	144.7 ± 0.8	**< 0.001**	**< 0.001**	**< 0.001**

GR [IU/g Hbg]	3.36 ± 0.20	3.48 ± 0.02	3.56 ± 0.02	3.69 ± 0.15	0.109	0.924	**< 0.05**

GR [IU/g protein]	92.00 ± 0.57	89.97 ± 1.05	93.15 ± 2.32	92.73 ± 1.23	**< 0.001**	**< 0.05**	0.184

MDA [mmol/L]	2.63 ( 2.62 – 2.65)	2.63 (2.61 – 2.65)	4.12 (4.01 – 4.14)	4.15 (4.13 – 4.65)	**< 0.001**	0.092	0.085

MDA [*μ*mol/g Hgb]	319.5 ± 13.4	315.0 ± 23.6	349.0 ± 6.9	402.3 ± 24.4	**< 0.001**	**< 0.01**	**< 0.01**

MDA [*μ*mol/g protein]	317.5 ± 12.5	339.2 ± 11.7	402.2 ± 6.7	315.3 ± 16.0	**< 0.001**	**< 0.001**	**< 0.001**

TAC [mmol/L]	3.40 ± 0.21	2.90 ± 0.05	1.67 ± 0.09	1.76 ± 0.12	**< 0.001**	**< 0.001**	**< 0.001**

p_inter_. – interaction between temperature and time							

Statistical significance was set at p < 0.05. Group −60/5: 1 min of WBC stimulation, 5 days, −60°C; Group −60/10: 1 min of WBC stimulation, 10 days, −60°C; Group −90/5: 1 min of WBC stimulation, 5 days, −90°C; Group −90/10: 1 min of WBC stimulation, 10 days, −90°C.

**Table 2:** Results of multiple comparisons in contrast analysis.

Temp.:	60 vs. 90		5 vs. 10	
Time:	5	10	60	90

Total SOD [NU/mL serum]	**< 0.001**	**< 0.001**	0.411	**< 0.01**

Total SOD [NU/mg Hgb]	**< 0.001**	**< 0.001**	**< 0.001**	0.644

Total SOD [NU/mg protein]	0.326	**< 0.001**	0.577	**< 0.001**

GPX [IU/g Hbg]	**< 0.001**	**< 0.001**	**< 0.001**	**< 0.001**

GPX [IU/mg protein]	**< 0.001**	**< 0.001**	0.645	**< 0.01**

CAT [IU/g Hbg]	**< 0.001**	**< 0.001**	0.407	**< 0.01**

CAT [IU/mg protein]	**< 0.001**	**< 0.001**	0.211	**< 0.001**

GST [IU/mg Hbg]	0.880	**< 0.001**	**< 0.001**	**< 0.01**

GST [IU/g protein]	**< 0.001**	**< 0.001**	**< 0.001**	**< 0.05**

GR [IU/g protein]	0.182	**< 0.01**	**< 0.05**	0.622

MDA [mmol/L]	0.236	**< 0.01**	0.996	0.094

MDA [*μ*mol/g Hgb]	**< 0.05**	**< 0.001**	0.679	**< 0.001**

MDA [*μ*mol/g protein]	**< 0.001**	**< 0.01**	**< 0.01**	**< 0.001**

TAC [mmol/L]	**< 0.001**	**< 0.001**	**< 0.001**	0.259

## Data Availability

The data used to support the findings of this study are available from the corresponding author upon request.
